# Corporate image as a mediator between service quality and customer satisfaction: difference across categorized exhibitors

**DOI:** 10.1016/j.heliyon.2019.e01307

**Published:** 2019-03-07

**Authors:** LiHsien Chien, ShuYi Chi

**Affiliations:** Department of Applied Economics, National Chung Hsing University, Taichung, Taiwan, ROC

**Keywords:** Business, Economics

## Abstract

Impact of service quality and corporate image on satisfaction and loyalty behavioral intention are explored by using the PLS-SEM (Partial Least Squares Structural Equation Modeling) analysis for the exhibition industry. Service quality has a significant enhancing effect on the corporate image of the trade exhibitions, and both have significant positive effects on exhibitor satisfaction. Also, the role of the image as a partial mediating variable between service quality and satisfaction is emphasized. Additionally, the results obtained from multi-group analysis also supported the hypothesis that corporate image and service quality bring different satisfaction responses in exhibitors of the different industry with 4 business sizes in the capital. In light of scale and types of the enterprises for trade shows market, price segmentation strategies should be offered to maintain satisfaction and loyalty from the SMEs. Service quality and image of the service-offering company are more emphasized by big enterprises in choosing the trade organizer. Multiple group analysis also considered categorizing the specific industrial enterprises. The organizer can apply the result to explore workable market strategies to meet the needs of business partners with different capital size. This research not only has avail for trade exhibition organizers but provides necessary theory-based research in the trade exhibition territory.

## 1 Introduction

Market is changing rapidly, and this is now the era of a flourishing globalized service industry that emphasizes the depth and breadth of service as well as service quality. Understanding what the customer values and expects of the service provided can aid companies in resource allocation and help them make improvements based on customer demand (Hsu et al., 2006). Therefore, the true understanding of what the customer perceives as high-quality service has become an important issue and requirement for the operations of every company or organization. Related research also points to the fact that how to improve service quality has become one of the main management nethods to increase customer loyalty and satisfaction (Su and Teng, 2018) and it is one of the critical factors influencing a company's success (Ahuja and Khamba, 2008).

Reichheld (1993) further indicated that, regardless of whether they are evaluating service quality or customer satisfaction, the primary aim for the business operators is always to grasp whether the product or service provided corresponds to the level of customer demand, so as to gain a competitive advantage in the marketplace with strong loyalty. Customer satisfaction can be viewed as a holistic assessment that represents the aggregate sum of the customer's subjective responses to the varying attributes of the product or service. In an exhibition, service quality and customer satisfaction are the primary success factors (Jin et al., 2012).

In the past, due to the flourish of emerging markets, it was relatively easy for companies to find and build new customer bases. As the market gradually become saturated, new customers are more difficult to find and costs are constantly rising, leading businesses to value long-term relationships with their customers (Solomon, 2010). Many researches apply SEM (Structural Equation Modeling) approach to study how the corporate image and service quality of the organization on the customers' satisfaction and the recipients' continuous intention to revisit; for example, gaming industry (Kim et al., 2017), hotel industry (Durna et al., 2015), beach resort hotel stay (Channoi et al., 2018), heritage tourism ((Wu and Li, 2017), and green marketing loyalty (Martinez, 2015). We consider the exhibition industry in our study by analyzing the major constructs from above. Multiple group analysis in the PLS-SEM is considered to categorize the specific industrial enterprises. The expositions organizer can apply the result to explore workable market strategies to meet the needs of business partners with different size.

Faced with huge food consumption demand and industrial scale, Taiwanese agricultural business are actively participating in various trade shows and striving for product exposure. This study targets exhibitors who have participated in past trade shows organized by the National Farmers' Association (NFA) in Taiwan as the research sample, to analyze the implications of service quality and corporate image on customer satisfaction and intention of repurchase behavior, as well as to gauge whether corporate image has a mediating effect between customer satisfaction and quality service. Considering the limite of sample size and multi-layer structure model characteristics, this study uses PLS-SEM model to perform construct estimation (Hair et al., 2016; Wetzels et al., 2009). We investigate to explore the impact and implications of the NFA's service quality and image on the exhibitors' satisfaction and the exhibitors' continuous intention to participate in future events; next, to examine the systematic correlation between service quality, corporate image, and exhibitors' satisfaction and behavioral intention, and the significance of the mediating effect of corporate image between service quality and satisfaction; third, to understand the effect of business size and industrial categories of the respondents to the system through the multi-group analysis.

## 2 Background

### 2.1 Service quality, corporate image, and customer satisfaction in the exhibition

Since the vigorous development of the global service industry, service quality has been playing an important role in creat customers value. The American Marketing Association defines service quality on their Dictionary page as “an area of study that has developed to define and describe how services can be delivered in such a manner as to satisfy the recipient.” Giving satisfactory services to partners may influence the customer's purchase decision of products. Related research also pointed out that the improvement of service quality is a key factor for business success (Ahuja and Khamba, 2008). Thus, firms can increase sales volume by offering good-quality service to increase their market share and the effectiveness of their service environment (Becerril-Arreola et al., 2017).

Due to three main characteristics: intangibles, heterogeneity, and inseparability, service quality is not easy to conceptualize (Parasuraman et al., 1988). The SERVQUAL(Service Quality Performance and Service Quality) scale proposed by Parasuraman et al. (1988) consists of five dimensions comprehensively: tangibility, reliability, assurance, responsiveness and empathy, widely used in service quality assessments in different research areas. Kim (2008) conducted research on South Korea's exhibitions, using the concept of SERVQUAL, and proposed and verified the measurement of the quality of trade show services and the performance of exhibitors.

Tor and Bodil (1998) found that if a company's service if good, it would only constitute a part of customers' overall assessment; conversely, if the customer is unfamiliar with the company's products or services, then the corporate image will become an important basis for decision making. Therefore, good service quality will lead customers who have prior experience to generate a positive image of comany, resulting in positive preferences. The positive image generated attracts new customers unfamiliar with the company to get to know the company or its products and services, eventually forming a transaction experience. Consequently, the corporate image generated by the services provided is crucial to the purchase decision process of existing and potential customers.

Corporate image plays a vital role in how service-oriented companies maintain customer loyalty (Harris and Goode, 2004). A favorable corporate image is considered a key factor influencing customer satisfaction and behavioral intentions (Faria and Mendes, 2013). Boulding (1956) defined the image as “a kind of subjective perception that refers to the viewpoint generated or perceived by certain people based on some fragmented experience or information.” The corporate image represents the operational capabilities and competitive advantage of a company; a good image creates trust in the mind of the customer. Fombrun (1995) also showed that companies with highly regarded develop some undisclosed assets that bring appealing competitive advantage by creating significant and consistent images. This relationship can also effectively enhance communication efficiency between both parties and can also achieve the strategic goals of stakeholders through economic or social interaction (Spyropoulou et al., 2010).

In business, corporate image is seen as a model that accumulates over time and continuously updated through consumer experience and also a stereotype constructed through the customer's experience of receiving services and consuming products (Bolton and Drew, 1991; Cronin and Taylor, 2008). When a company image is perceived receipents will consider the quality of products and services provided by the said company is high, thus making it easy to form a high customer satisfaction, and they will be more willing to accept the company's services and buy the company's products (Goldberg and Hartwick, 1990; Hsu et al., 2006). A superior corporate image cannot only encourage customers to choose their services, but also improve their satisfaction with the company (Faria and Mendes, 2013).

A Company image is the result of many factors which influence customers and some of these factors. As NFA plays a market companies role, this study adopted the corporate character scale method which was developed and tested by Davies et al. (2004). The corporate image involves enterprise, competence, and informality, which represent in following facets: modernity, adventure, boldness, conscientiousness, drive, technocracy and simple (Maťová et al., 2015; Davies et al., 2004).

Satisfaction is a cognitive evaluation process in which consumers compare actual product performance with their prior expectations (Parasuraman et al., 1993; Westbrook, 1980) and is used to assess the most positive response in the customer's experiential value (Oliver, 1997). Satisfaction is also a dynamic and concrete concept (Cronin and Taylor, 1994; Giese and Cote, 2000), which is influenced by service quality, product quality, price, and contextual and personal factors (Zeithaml and Bitner, 2000). It can be divided into: specific transaction, cumulative, cognitive, and affective (Anderson et al., 1994). As for customer satisfaction can be viewed as a holistic assessment that represents the aggregate sum of the customer's subjective responses to the varying attributes of the product or service (Fornell, 1992) it seeks to increase customer loyalty, thereby creating better operational performance (Gronholdt et al., 2000; Martensen et al., 2000) and showing that positive or negative corporate image is closely linked to customer satisfaction. From the psychological level, if the actual result felt by the exhibitor is better than expected, it will produce a satisfactory feeling, otherwise it will be dissatisfied; on the economic level, the customer satisfaction depends on the value obtained, that is, The ratio of service quality or commercial benefit to the cost of participation compared to exhibitors (Parasuraman et al., 1988).

## 3 Hypothesis

According to the research in the exhibition, service quality and customer satisfaction are the primary factors in building an exhibition success (Jin et al., 2012). It also becomes one of the main management methods to improve customer loyalty and satisfaction (Su and Teng, 2018). Simply put, the organizer's image as well as the products and services provided have a direct and close relationship with the exhibitors' satisfaction response; hence, this study puts forth the following hypotheses:

H1 Corporate Image (CI) has a significant and positive effect on exhibitor satisfaction (CS).

H2 Service Quality (SQ) has a significant and positive effect on Corporate Image (CI).

H3 Service Quality (SQ) has a significant and positive effect on Customer Satisfaction (CS).

### 3.1 Customer satisfaction and behavioral intention in the exhibition

The concept of behavioral intention originated from the attitude theory proposed by Engel et al. (1995), which refers to the behavioral tendencies possibly assumed by consumers after receiving services or purchasing products in the company that provided the said product or service. A pleasant consumer experience created by the provider's services will increase consumer trust in the firm (Sirdeshmukh et al., 2002). Customers' behavioral intention can demonstrate their evaluation and feelings toward an environmental experience process, thus affecting their attitude and future purchase intention, including the possibility of giving a referral to others, as well as their own repurchase intention. The responses reflecting customer satisfaction can be seen as an indicator influencing their business loyalty.

According to Zeithaml et al. (1996), research on the influence of service quality on customer behavior can be categorized as positive and negative behavioral intentions. When a customer's opinion of a company's products or services reaches a certain level, signifying increased satisfaction, their response will be to increase their purchase volume and praise the company, which then gives rise to special preferences that generates positive behavioral intention, thus enhancing future interactions between the customers and the company, and their behavioral intentions are expressed through loyalty. Conversely, if customers reduce or stop consuming a company's products or services altogether, that is a negative behavioral intention toward the company, or satisfaction declining, the response would be to stop using the company's services or purchasing its products and no longer purchase or recommend others to use its services.

In the current business climate where it is difficult to build new customer bases, the cost of developing new customer groups often exceeds that of maintaining the existing ones (Solomon, 2010). Westbrook (1987) thought that satisfaction is generally regarded as a mediating variable in consumer repurchase intention. The better a firm's service, the greater will be customer satisfaction, which further affects their behavioral intention, resulting in a positive development (Zeithaml et al., 1996; Boulding et al., 1993; Gronholdt et al., 2000). Only when firms understand their customers' behavioral intentions and make an effort to realize the expectations of goods or services as well as induce positive responses in the customers, will there be a long-term and sustainable willingness to make transactions. Firms can then have sustainable operations (Kim et al., 2017). Hence, this provides a valuable reference for companies in their long-term operations (Wu and Li, 2017; Martinez, 2015).

As previously discussed, customer satisfaction is the emotional factor of the customers' experience after their purchase. Therefore, customers' perception of service quality and corporate image affects their evaluation of the overall satisfaction and their subsequent payment intention. Service quality is the prerequisite variable of customer satisfaction. Essentially, service quality leads to satisfaction, which in turn generates behavioral intention. This means that there is a higher probability that satisfied service users will willingly consider repurchasing or reusing a service than those who express dissatisfaction (Valaei and Baroto, 2017). For this reason, hypothesis four (4) is established as follows:

H4 Customer Satisfaction (CS) has a significant and positive effect on customer's post-event behavioral intention (BI).

## 4 Materials & methods

### 4.1 PLS-SEM assessment

Studies on marketing issues have been using quasi-standard structural equation modeling (SEM) to analyze and evaluate the results as a common practice in recent years (Hulland, 1999; Hair et al., 2012). There are two methods with which to estimate the derivation of model parameters—utilizing the CB-SEM of maximum likelihood estimation (Hair et al., 2010) and the PLS-SEM of ordinary least squares. In contrast to CB-SEB, which emphasizes the calculation of estimated value and measurement item covariance under the requisite conditions of large sample size and normal distribution, PLS-SEM estimates the value of the latent variables through linear combination of relevant observed variables. Therefore, PLS-SEM has a more extensive usage and fewer data restriction attributes and has become an increasingly prevalent analytical tool in market research and social sciences (Chin, 2010; Hair et al., 2012).

### 4.2 Questionnaire and pre-test

There are four constructs in this study. The corporate image construct includes three observed variables such as NFA image, advertising image, and NFA reputation. The service quality dimension includes five variables—tangibility, reliability, assurance, responsiveness, and empathy. The customer satisfaction construct includes three variables—payment of expenses, exhibitor performance, and overall satisfaction. The behavioral intention construct consists of two variables—loyalty and intention to pay. The questionnaires employed the Likert five-point scale, where 1 indicates strongly disagree and 5 indicates strongly agree. The draft of the questionnaire was first distributed to 10 experts who were familiar with trade show activities to conduct pre-tests to ensure that the corresponding questionnaires were discriminative before conducting the actual survey.

### 4.3 Sampling design and data collection

According to the NFA's data, a total of 110 exhibitors participated in the trade fairs organized by the NFA during 2011–2016 with approximately 160 personnel who were invited to fill out the questionnaire on the designated web page containing this research via email or social networks. A total of 150 questionnaires were sent out from July to August 2016, out of which 113 valid questionnaires were extracted after eliminating unqualified samples, with a qualified completion rate of 75.3% (see Table 1).

**Table 1:** The reference of research dimension and hypothesis.

Dimension	Facet	Reference
Service quality	Tangibility; Reliability; Assurance; Responsiveness And Empathy	Parasuraman et al. (1988); Kim (2008)
Corporate image	Enterprise; Informality; Competence	Cronin and Taylor (2008); Spyropoulou et al. (2010); Mať'vá et al. (2015)
Customer satisfaction	Expenses; Performance; Overall Expectation	Kotler and De Bes (2003); Parasuraman et al. (1988); Anderson et al. (1994)
Behavioral intention	Loyalty; Payment decision	Martensen et al. (2000); Parasuraman et al. (1988)

Research hypothesis		

*H1: Corporate Image (CI) has a significant and positive effect on exhibitor satisfaction (CS)*		Hsu et al. (2006); Faria and Mendes (2013)
*H2: Service Quality (SQ) has a significant and positive effect on Corporate Image (CI)*		Faria and Mendes (2013); Solomon (2010)
*H3:Service Quality (SQ) has a significant and positive effect on Customer Satisfaction (CS)*		Su and Teng (2018); Jin et al. (2012)
*H4:Customer Satisfaction (CS) has a significant and positive effect on customer's post-event behavioral intention (BI).*		Wu and Li (2017); Kim et al. (2017); Durna et al. (2015)

Of the vendors who responded, 67 were male and 46 were female. The 31–40-year-old demographic was the largest and accounted for 32.7% of the sample and included a total of 37 persons; 57 respondents were university-educated (professionals), which was the majority and accounted for 50.4% of the sample; 73 persons were high-level executives. Most of the exhibitors held a capital of under NTD$ 5 million (approximately USD$ 160,000). Vendors in the food processing industry accounted for the majority (37 persons), followed by farmers' associations (30 persons) and vendors in the tea industry (22 persons). The respondent demographic is summarized in Tables 2 and 3, as shown below.

**Table 2:** Respondent identity demographic of samples (N = 113).

Categories	Characteristics	Frequency	Percent
Gender	Male	67	59.3
	Female	46	40.7
Age	21–30	8	7.1
	31–40	37	32.7
	41–50	35	31.0
	51–60	23	20.4
	61 and above	10	8.8
Education	Diploma	36	31.8
	Bachelor Degree	57	50.4
	Master Degree and above	20	17.7

**Table 3:** Company representative demographic of samples (N = 113).

Categories	Characteristics	Frequency	Percent
Working Position	General manager	33	29.2
	Manager	39	34.5
	Sale Specialist	18	15.9
	Salesman	23	220.4
Industry	Food processing	37	32.7
	Rural organization	30	26.5
	Tea industry	22	19.5
	Grocery	24	21.3
Capital Scale	5 million NTD and below	50	44.2
	5–10 million NTD	18	15.9
	10 million and above	24	21.2
	No response	21	18.6

Average exchange rate between NTD and USD is 30:1 as of 2018.

## 5 Results

### 5.1 Convergent validity and reliability

In this study, the PLS-SEM reflective is constructed consisting of four latent variables: corporate image, service quality, customer satisfaction, and behavioral intention, and the model estimation follows the indicators proposed by previous researchers (Chin, 2010; Götz et al., 2010).

The statistical results show that factor loadings of all variables are greater than 0.7 only with one exception which is slightly less than 0.7 (CI12 for 0.697), conforming to the conditional requirements (Hair et al., 2012; Hulland, 1999) (see Table 4). The internal reliability Cronbach's alpha values are between 0.708 and 0.901, while the CR (Composite Reliability) values are between 0.755 and 0.949, showing that all latent variables in this study have a high degree of internal consistency and reliability. All tests conform to the required conditions, indicating that each variable is suitable for follow-up analysis and verification. The AVE (average variance extracted) values of the four latent variables in the research model are corporate image 0.391, service quality 0.352, consumer satisfaction 0.409, behavioral image 0.559, and all values are greater than 0.3 and are thus on the whole in line with the recommended assessment values for the latent variables and AVE. The verification results are acceptable, showing that there is sufficient explanatory power in the measurement variables for the corresponding latent variables to be used in analysis (Bagozzi and Yi, 1988; Fornell and Larcker, 1981; Hair et al., 2006).

**Table 4:** Construct reliability and validity.

Construct	Item	Loading	Cronbach's α	C.R.	AVE
	Corporate Image (CI)		0.708	0.804	0.391
Enterprise	The NFA has good word-of-mouth compared with other trade-show organizers (M12)	0.856	0.368	0.755	0.609
	NFA provides exhibitors a channel for feedback and suggestions (M14)	0.697			
Imformality	NFA-organized trade shows can increase the visibility of my products (M22)	0.818	0.378	0.762	0.616
	NFA-organized trade shows can enhance the international image of Taiwan's agricultural products (M23)	0.750			
Competence	NFA-organized events are beneficial and trustworthy (M31)	0.858	0.866	0.919	0.791
	The C/P Ratio of NFA events is appealing (M33)	0.944			
	The NFA is my preferred event organizer (M34)	0.863			
	Service Quality (SQ)		0.868	0.891	0.352
Tangibility	The NFA arranges convenient food and accommodations around the show (Q12)	0.911	0.896	0.924	0.711
	The NFA provides exhibition locations on the show floor for optimal product display (Q13)	0.880			
	The details arranged by NFA are consistent with the theme of the show (Q15)	0.752			
	NFA organized shows have free-flowing visitors aisles (Q16)	0.752			
	NFA staff members look neat, tidy, and relaxed (Q17)	0.907			
Reliability	NFA trade show events are very safe for product exhibition (Q21)	0.759	0.727	0.846	0.647
	The NFA can ensure individual exhibitors' safety (Q23)	0.852			
	The NFA carefully evaluates products for exhibition (Q24)	0.799			
Assurance	NFA service personnel are very professional (Q31)	0.821	0.775	0.855	0.598
	NFA service personnel are polite (Q33)	0.812			
	NFA service personnel deserve my trust (Q32)	0.741			
	NFA service personnel can solve problems (Q34)	0.713			
Responsiveness	NFA service personnel are effective administrators (Q41)	0.915	0.739	0.883	0.791
	The entire NFA team's internal communication is excellent (Q44)	0.862			
Empathy	Generally, there is no preferential treatment in the service the NFA offers (Q53)	0.958	0.900	0.952	0.909
	I do not receive bureaucratic service from the NFA (Q54)	0.949			
	Customer Satisfaction (CS)		0.788	0.844	0.409
Expenses	The logistics fees the NFA charges are reasonable (S12)	0.703	0.667	0.817	0.601
	I feel the utilities charges in the show are reasonable (S13)	0.735			
	I feel the fees for part-time student workers are reasonable (S14)	0.876			
Performance	Participating in NFA shows can develop potential customer bases (S22)	0.704	0.720	0.804	0.644
	Participating in NFA shows can get me orders from existing customers (S24)	0.856			
	Participating in NFA shows can get me new-customer orders (S25)	0.839			
Overall Expectation	The services provided at the show meet my every expectation (S31)	0.928	0.841	0.927	0.863
	The level of service provided at trade show event comes close to what I envisioned when being solicited to join the show (S32)	0.930			
	Behavioral Intention (BI)		0.901	0.919	0.559
Loyalty	I will give a good referral to my peers who are being solicited to join the NFA trade show events (B11)	0.866	0.914	0.936	0.746
	I will invite my peers to join NFA-organized trade show events (B12)	0.889			
	I hope to participate in the next NFA-organized trade show event (B13)	0.915			
	I will make all NFA-organized trade show events my first choice (B14)	0.830			
	If anyone asks me to recommend a trade show to join, I will recommend the NFA events (B15)	0.813			
Payment decision	If the NFA charges a service fee in connection with the shows, I will be willing to pay to join (B21)	0.920	0.928	0.949	0.824
	Even though the fees the NFA charges in the shows are higher than other companies, I am still willing to pay (B22)	0.950			
	Due to the fact that I can receive a great deal of benefit from NFA-organized events, I am willing to pay more to participate (B23)	0.836			
	Even service costs more and caused the costs to increase, I will still accept the service (B24)	0.922			

Notes: A denotes the NFA of the ROC, CR = Composite Reliability, AVE = Average Variance Extracted.

Data Source: Results of this research.

### 5.2 Discriminant validity

In assessing discriminant validity, the pertinent constructs must conform to the tests of cross-loadings and Fornell–Larcker Criterion (Chin, 2010). The results of this study show that all items met expectations, and individual items were separated into corresponding constructs (see Table 5) (Chin, 2010, 1998; Fornell and Larcker, 1981). Taking variable B11 for example, the factor load values of the four constructs are 0.717, 0.278, 0.409, and 0.409, respectively. According to the maximum load classification, this variable has the highest load value (0.717) for the behavioral intention construct, which shows that there is high explanatory power in behavioral intention, and therefore, it is classified in this construct. This also confirms the original setup. Through the examination of Fornell–Larcker criterion (Fornell and Larcker, 1981; Götz et al., 2010), the four major constructs in this study meet the above-mentioned criteria, so the discriminant validity used in this study can be confirmed.

**Table 5:** Discriminant validity and cross-loadings analysis.

first-order constructs	Fornell-Larcker Criterion			
	CI	CS	SQ	BI
CI	**0.625**			
CS	0.600	**0.639**		
SQ	0.486	0.633	**0.593**	
BI	0.490	0.566	0.600	**0.748**

items	Cross-Loadings Analysis			

M12	**0.563**	0.339	0.335	0.364
M14	**0.405**	0.309	0.176	0.195
M22	**0.429**	0.36	0.2	0.125
M23	**0.373**	0.235	0.205	0.153
M31	**0.766**	0.398	0.339	0.306
M33	**0.852**	0.475	0.376	0.463
M34	**0.789**	0.451	0.407	0.407
Q12	0.469	0.588	**0.791**	0.526
Q13	0.417	0.577	**0.789**	0.417
Q15	0.462	0.501	**0.684**	0.537
Q16	0.363	0.471	**0.636**	0.391
Q17	0.4	0.544	**0.8**	0.459
Q21	0.319	0.327	**0.531**	0.379
Q23	0.448	0.449	**0.692**	0.561
Q24	0.241	0.289	**0.602**	0.37
Q31	0.119	0.268	**0.482**	0.174
Q32	0.154	0.212	**0.612**	0.241
Q33	0.128	0.206	**0.461**	0.22
Q34	0.166	0.207	**0.477**	0.169
Q41	0.164	0.324	**0.408**	0.239
Q44	0.101	0.31	**0.324**	0.194
Q53	0.152	0.234	**0.482**	0.296
Q54	0.154	0.216	**0.439**	0.218
S12	0.289	**0.481**	0.243	0.122
S13	0.303	**0.555**	0.511	0.331
S14	0.328	**0.721**	0.564	0.458
S22	0.412	**0.614**	0.456	0.517
S24	0.365	**0.737**	0.558	0.428
S25	0.283	**0.739**	0.414	0.392
S31	0.623	**0.605**	0.204	0.278
S32	0.487	**0.615**	0.206	0.278
B11	0.278	0.409	0.409	**0.717**
B12	0.339	0.482	0.516	**0.76**
B13	0.395	0.499	0.604	**0.793**
B14	0.429	0.459	0.562	**0.789**
B15	0.296	0.456	0.453	**0.719**
B21	0.386	0.371	0.368	**0.739**
B22	0.403	0.409	0.411	**0.799**
B23	0.362	0.384	0.36	**0.678**
B24	0.405	0.329	0.329	**0.727**

### 5.3 Goodness of model fit

**Fig. 1:**
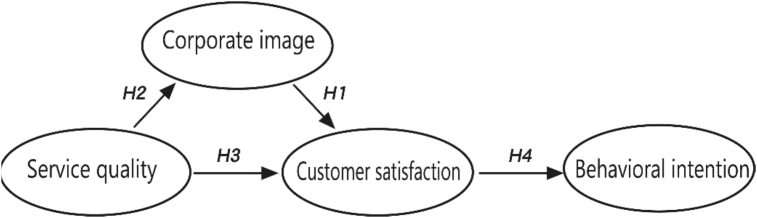
Research framework.

PLS models can be assessed through tests of model fit (Dijkstra and Henseler, 2015). The GoF (Goodness of fit) index serves as baseline values for validating the PLS model globally (Cohen, 1988; Tenenhaus et al., 2005; Wetzels et al., 2009). It can be calculated by the geometric mean of the average AVE and the average $R^{2}$ for endogenous constructs (see Fig. 1). Its value should range between 0 and 1. Wetzels et al. (2009) proposed that the cut-off values for different thresholds are GoFsmall = 0.1, GoFmedium = 0.25, and GoFlarge = 0.36, respectively. It is apparent from Table 3 that the AVE of the first four constructs in order are 0.391, 0.352, 0.409, and 0.559, respectively, resulting in a geometric mean of 0.421; the $R^{2}$values for corporate image, customer satisfaction, and behavioral intention constructs are 0.236, 0.513, and 0.320, respectively, resulting in an average value of 0.356; therefore, the goodness of fit (GoF) value can be calculated by the following formula:

$$GoF=\sqrt{\bar{AVE^{G}}\times R^{2}}=\sqrt{0.421\times 0.356}=0.387$$

The result is 0.387, which conforms to the test standards, showing that the overall model has goodness of fit and there are indeed significant relationships between corporate image, customer satisfaction, and behavioral intention.

## 6 Analysis

### 6.1 Results of hypotheses testing

**Fig. 2:**
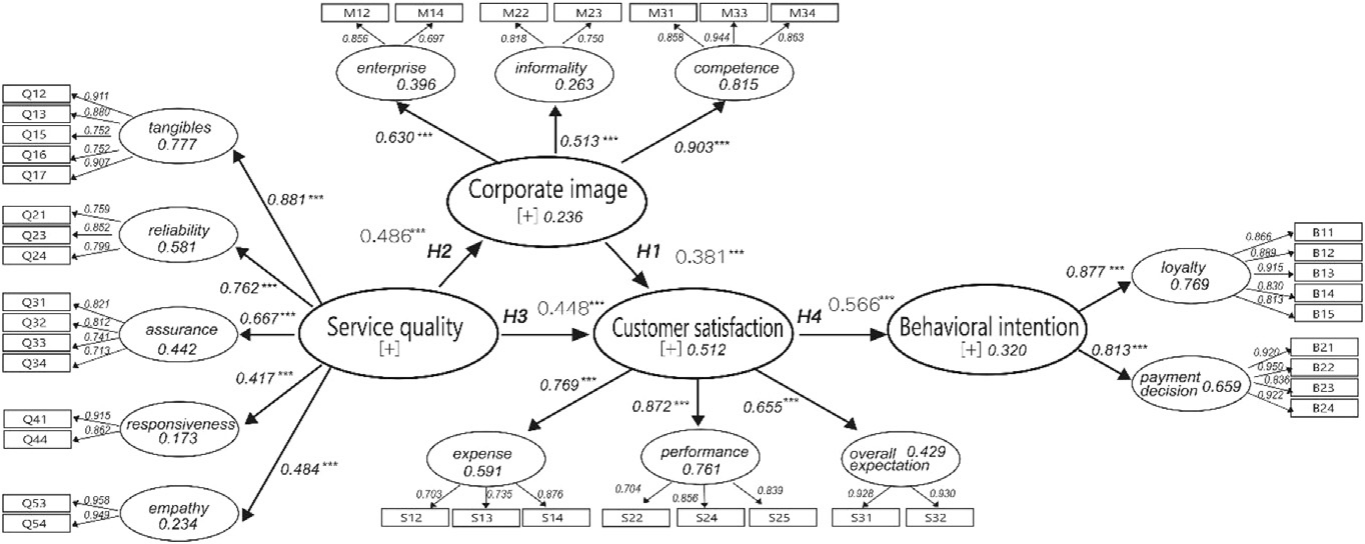
PLS results.

PLS models can be assessed through measures of model fit based on bootstrapping (Henseler et al., 2015; Dijkstra and Henseler, 2015). Bootstrapping is a non-parametric procedure with which to estimate the statistical significance of the path coefficient results of the measure a model and the explanatory power of the construct ($R^{2})$ (Hair et al., 2011). In this study, after repeating the calculation 5,000 times using the bootstrapping method, the explanatory power values ($R^{2})$of corporate image, customer satisfaction, and behavioral intention constructs are 23.6%, 51.2%, and 32.0%, respectively. This shows that corporate image has a significant positive effect on customer satisfaction (β = 0.381, p < 0.001), service quality has a significant positive effect on both corporate image (β = 0.486, p < 0.001) and customer satisfaction (β = 0.448, p < 0.001), and customer satisfaction has a significant positive effect on behavior intention (β = 0.566, p < 0.001). Therefore, the four hypotheses, H1, H2, H3, H4 in this study are valid (see Table 6). The structure of PLS-SEM results is shown in Fig. 2.

**Table 6:** Structure model hypothesis testing.

Hypothesis & Path	Coefficients	Mean	S.D	T Statistics	P Values	Result
H1: CI → CS	0.381	0.377	0.073	5.203***	0.000	Supported
H2: SQ → CI	0.486	0.492	0.083	5.858***	0.000	Supported
H3: SQ → CS	0.448	0.454	0.072	6.197***	0.000	Supported
H4: CS → BI	0.566	0.568	0.082	6.895***	0.000	Supported

Note: S.D. = standard deviation; significance level:^∗∗∗^P < 0.001 (T statistics≧3.29); 5,000 bootstrap samples.

### 6.2 The mediating effect of corporate image on customer satisfaction

It is evident that both service quality and corporate image have a positive and significant effect on satisfaction. When further considering how the NFA's corporate image affects the exhibitors' satisfaction with the NFA's service, corporate image can be set as the mediating variable between service quality and satisfaction to examine whether there is a mediating effect. Fig. 2 shows that the direct-effect value of service quality on corporate image and of corporate image on service satisfaction is 0.486 and 0.381, respectively. Then, with image as the mediating variable, the indirect effect of service quality on satisfaction can be calculated as the product of the front-end and back-end values resulting in 0.185 (= 0.486*0.381); the sum of the direct effect (0.448) and indirect effect (0.185) of service quality to satisfaction equals the total effect (0.633). Hence, the impact of the service quality factor on the customer satisfaction variance consists of not only the 44.8% influence of the service quality factor itself but also approximately 18.5% influence generated indirectly through the corporate image construct.

According to the requirement for the mediator by Baron and Kenny (1986), the paths between the three variables must be statistically significant and the Sobel test (Sobel, 1982; Anderson et al., 1994) is utilized to test indirect effect and use the results to significantly support that the effect exists. The results show that the VAF (variance accounted for) value is 29.2% (= 0.185/0.633), which is less than 80%, thereby fulfilling the condition of establishing partial mediator (Hair et al., 2014), and thus, a partially mediated relationship is established. In other words, service quality itself not only directly affects satisfaction but also indirectly affects customer satisfaction through the mediating effect of corporate image.

### 6.3 Multigroup analysis

This study follows the multi-group analysis to decode a certain construct system, whether the mediating effect between service quality and customer satisfaction will be influenced by the industrial category and the business size of the respondent's company. The respondents' industrial attributes are categorized into four groups, food processing (FP), tea (TE), rural organizaiton (RO), and grocery (GC). Based on statement of the OECD data book, enterprises can be classified in different categories according to their size; and since the number of people employed is unavailable in this study, capital scale of the respondents is considered to the proxy for measurement. Small and medium-sized enterprises (SMEs) are the major business size in Taiwan. All 113 respondents are divided into large enterprises with more than NT$10 million scale in capital, medium-sized enterprises (between NT$10 million and NT$5 million), small enterprises (below NT$5 million), and the group of no response. Path coefficient (β) of each group are estimated separately.

Table 7 shows that the customer satisfaction was significantly affected by service quality (SQ→CS) and the mediating effect of the NFA's image (CI) in the FP industry exists at VAF = 24%. Satisfaction of the TE industry respondents was directly affected by the NFA's image (CI →CS), while image was significantly affected by service quality (SQ→CI). The behavioral intertion of all respondents are positive and strong influenced by the satisfaction they received and conformed (CS→BI) (Diallo et al., 2018).

**Table 7:** Path analysis results for different industrial groups.

Industry	Food Processing (n = 37)		Tea Industry (n = 22)		Rural Organizaiton (n = 30)		Grocery (n = 24)	
	β	T-value	β	T-value	β	T-value	β	T-value
H1: CI →CS	0.317	2.046*	0.510	2.173*	0.361	1.646	0.431	1.861
H2: SQ→CI	0.482	2.462*	0.485	2.148*	0.516	3.984***	0.491	1.881
H3: SQ→CS	0.467	2.840**	0.277	1.011	0.547	2.788**	0.512	2.732**
H4: CS→BI	0.525	2.924**	0.746	4.969***	0.539	3.258**	0.710	5.639***
Mediation Effect	β = 0.619(VAF = 24%)		No		No		No	

Note: *P < 0.05(T≧1.96); **P < 0.01(T≧2.58); ***P < 0.001(T ≧3.29).

As for capital scale, for the respondents serving companies with large enterprises, the relationship between four latent constructs is statistically significant and consistent with expectations, the NFA's image also support a partially mediating effect on satisfaction. Those large exhibitors show their attentions on service quality and corporate image of the event organizer and significantly support the influence of the two constructs above on the satisfaction of the trade show. However, for respondents serving companies with samall scale, only the relationship between satisfaction and subsequent behavioral intentions remains positively significant (see Table 8). This result indicats that when the respondents come from small companies, their satisfaction to the exhibitions is not significantly affected by the quality of services NFA's provided and their image, neither. The follow-up behavioral intentions are still significantly affected by key factors in the satisfaction construct such as expenses, organizer performance, and overall satisfaction.

**Table 8:** Path analysis results for business with different scale.

Business Size	Large (n = 24)		Medium (n = 18)		Small (n = 50)		No response (n = 21)	
	β	T-value	β	T-value	β	T-value	β	T-value
H1: CI →CS	0.527	3.299^**∗∗**^	0.236	0.825	0.366	1.158	0.552	2.150*
H2: SQ→CI	0.500	2.898**	0.774	2.766*	0.318	1.721	0.566	2.816**
H3: SQ→CS	0.436	2.594**	0.515	1.670	0.405	1.935	0.486	2.134*
H4: CS→BI	0.498	2.363	0.586	2.868*	0.593	4.160***	0.721	3.701***
Mediation Effect	β = 0.699 (VAF = 37%)		No		No		β = 0.798 (VAF = 39%)	

Note: *P < 0.05 (T≧1.96); **P < 0.01 (T≧2.58); ***P < 0.001 (T ≧3.29).

Multi-group analysis results show that the difference between the service industry and the company's capital scale will significantly affect the clinents' satisfaction of the NFA's handling of the trade fair's activities. Therefore, the fair organizer should design trade shows based on the requirements of the different exhibitors in regards to image and service quality so as to meet the needs of all parties. Only then would it be possible to effectively improve performance and achieve higher overall benefits.

## 7 Discussion & conclusion

This study surveys the representatives of the exhibitors that participated in overseas trade shows organized by the NFA as research subjects. Through a survey with the 113 exhibitors, this research presumes the implications of organizer's service quality and corporate image on the exhibitors' satisfaction and subsequent behavioral intentions.

The statistical results show that the scale indicators used in this study conform to the standards set forth within various assessment criteria, as they have both good reliability and validity and the model fit is ideal. Exhibitors' satisfaction was significantly affected by the service quality of the organizer. This is in line with the findings of many articles which supports the significant positive relationship between service quality and customer satisfaction. Zeithaml et al. (1996) and Anderson et al. (1994) Using the trade show organized by the NFA as an example, our results shows that the service quality and image of the organizer did exhibit a significant positive relationship with exhibitors' satisfaction.

A significant positive effect Service quality is on the corporate image is confirmed. Yilmaz and Ari (2017) found the same result for the high-speed rail service sector in Turkey. Priporas et al. (2017). The tangibility, reliability, handling capabilities, empathic and amiable service content provided by the NFA improved the image of the NFA in the minds of exhibitors, whose satisfaction and subsequent behavioral intentions had significant positive effects. According to the analysis, if satisfaction, which is measured by the two constructs of service satisfaction and exhibitor performance, increases, it will affect exhibitors' post-event corresponding loyalty and financial evaluation response. In other words, if the exhibitors are satisfied with the service content provided by the NFA, and there is a significant improvement in exhibitors' trade show performance, then their subsequent intention to participate in trade shows organized by the NFA will manifest itself as increased loyalty. In addition, they will still be willing to participate, even if they must incur higher costs. It is shown that the participants are also willing to pay higher fees for the services obtained.

In this study, we also analyze whether corporate image has a mediating effect between service quality and exhibitor satisfaction. The statistical results support the fact that the corporate image has a partial mediating effect on service quality and exhibitor satisfaction, implying that the level of exhibitor satisfaction is not only affected by the variables of service quality and corporate image individually. This is consistent with the findings of several studies (Goldberg and Hartwick, 1990), which support the impact of the positive corporate image on satisfaction and purchase intention.

Results of this study also support that large size enterprises tend to cooperate with exhibition organizers with a positive and beneficial image in the market to strengthen the effect of service quality to their satisfaction and, later, the behavioral intention in loyalty (Nguyen and Leblanc, 2001). If the scale of companies implies more strong needs for business exposure to products by participating in the exhibition but with limited loyal intention, the NFA may implement tailored services to approach to these companies. This marketing strategy may effectively enhance customer satisfaction and the loyalty of customers. To SMEs with limited resources in presenting their products in the exhibitions, the NFA should focus on improving their satisfaction in terms of reasonable fee, operating performance, and overall expectation (Giovanis et al., 2014). With good satisfaction, the connection to loyalty is apparent for SMEs.

Based on the above research and discussion, the NFA has indeed established a beneficial image among the exhibitors through the excellent and comprehensive service provision and planning, as well as its image, after many years of trade show-organizing activities and services. After that, the NFA should have a clear understanding that service quality is the key to generating satisfaction and post-show behavioral intentions. Therefore, the NFA should maintain a good corporate image, provide good quality services in accordance with the company's characteristics, and meet exhibitors' exhibiting needs to increase the revenue and exhibitors' loyalty.

## Declarations

### Author contribution statement

LiHsien Chien, ShuYi Chi: Conceived and designed the experiments; Performed the experiments; Analyzed and interpreted the data; Contributed reagents, materials, analysis tools or data; Wrote the paper.

### Funding statement

This research did not receive any specific grant from funding agencies in the public, commercial, or not-for-profit sectors.

### Competing interest statement

The authors declare no conflict of interest.

### Additional information

Data associated with this study has been deposited at 4TU.Centre for Research Data under the accession number https://doi.org/10.4121/uuid:14e902b9-5c83-4f73-a342-49bb34959d43.
